# A collection of split-Gal4 drivers targeting conserved signaling ligands in *Drosophila*

**DOI:** 10.1093/g3journal/jkae276

**Published:** 2024-11-21

**Authors:** Ben Ewen-Campen, Neha Joshi, Ashley Suraj Hermon, Tanuj Thakkar, Jonathan Zirin, Norbert Perrimon

**Affiliations:** Department of Genetics, Blavatnik Institute, Harvard Medical School, Harvard University, Boston, MA 02115, USA; Department of Genetics, Blavatnik Institute, Harvard Medical School, Harvard University, Boston, MA 02115, USA; Department of Genetics, Blavatnik Institute, Harvard Medical School, Harvard University, Boston, MA 02115, USA; Department of Genetics, Blavatnik Institute, Harvard Medical School, Harvard University, Boston, MA 02115, USA; Department of Genetics, Blavatnik Institute, Harvard Medical School, Harvard University, Boston, MA 02115, USA; Department of Genetics, Blavatnik Institute, Harvard Medical School, Harvard University, Boston, MA 02115, USA; Howard Hughes Medical Institute, Boston, MA 02115, USA

**Keywords:** Drosophila, cell signaling, signaling pathways, ligands, split-Gal4, reporter lines

## Abstract

Communication between cells in metazoan organisms is mediated by a remarkably small number of highly conserved signaling pathways. Given this small number of signaling pathways, the existence of multiple related ligands for many of these pathways represents a key evolutionary innovation for encoding complexity into cell–cell signaling. Relatedly, crosstalk between pathways is another critical feature, which allows a modest number of pathways to ultimately generate an enormously diverse range of outcomes. It would thus be useful to have genetic tools to identify and manipulate not only those cells that express a given signaling ligand but also those cells that specifically coexpress pairs of signaling ligands. We present a collection of split-Gal4 knock-in lines targeting many of the ligands for highly conserved signaling pathways in *Drosophila* (Notch, Hedgehog, fibroblast growth factor (FGF), epidermal growth factor (EGF), transforming growth factor *β* (TGF*β*), Janus kinase/signal transducer and activator of transcription (JAK/STAT), Jun kinase (JNK), and platelet-derived growth factor (PDGF)/vascular endothelial growth factor (VEGF)-related receptor (PVR). We demonstrate that these lines faithfully recapitulate the endogenous expression pattern of their targets and that they can be used to identify cells and tissues that coexpress pairs of ligands. As a proof of principle, we demonstrate that the 4th chromosome TGF*β* ligands *myoglianin* and *maverick* are broadly coexpressed in muscles and other tissues of both larva and adults and that the JAK/STAT ligands *upd2* and *upd3* are partially coexpressed from cells of the midgut following gut damage. Together with our previously collection of split-Gal4 lines targeting the 7 Wnt ligands, this resource allows *Drosophila* researchers to identify and genetically manipulate cells that specifically express pairs of conserved ligands from nearly all the major intercellular signaling pathways.

## Teaser

Intercellular signaling in animals by a small number of highly conserved signaling pathways, and most of these pathways can be activated by multiple closely related ligands. Here, Ewen-Campen *et al*. present a collection of split-Gal4 lines targeting the ligands of the major signaling pathways in *Drosophila*. These transgenic lines allow researchers to identify and genetically manipulate cell populations which specifically express nearly any pair signaling ligands for the major signaling pathways in the fruit fly.

## Introduction

Coordinated activity between cells in multicellular organisms requires the activity of intercellular signaling pathways, where signaling cells generate ligands that are received and interpreted by cells which express relevant receptors. A relatively small number of intercellular signaling pathways are extraordinarily highly conserved across animal evolution; these pathways include Notch, Hedgehog, fibroblast growth factor (FGF), epidermal growth factor (EGF), Wnt/Wingless, transforming growth factor *β* (TGF*β*), Janus kinase/signal transducer and activator of transcription (JAK/STAT), Jun kinase (JNK), platelet-derived growth factor (PDGF)/vascular endothelial growth factor (VEGF)-related receptor (PVR), and others (Perrimon *et al*. 2012; Housden and Perrimon 2014).

Despite the relatively small number of highly conserved signaling pathways, an enormous complexity of outcomes is possible. Among the many features that enable complexity in metazoan signaling is the fact that there are often multiple closely related ligands capable of activating a given pathway (Perrimon *et al*. 2012; Housden and Perrimon 2014). In the *Drosophila* genome, for example, while there is only a single characterized ligand for the Hedgehog pathway [*hedgehog (hh)*] and for the JNK pathway [*eiger (egr)*], there are 2 paralogous ligands for the Notch receptor (*Delta* and *Serrate*), 3 FGF ligands (*bnl*, *ths*, and *pyr*), 3 JAK/STAT ligands (*upd1*–3), 3 PVR ligands (*Pvf1–3*), 4 EGF agonistic ligands (*spitz*, *gurken*, *Keren*, and *vein*), and 7 ligands each for Wnt and TGF*β* signaling (Nusse 2001; Upadhyay *et al*. 2017). The fact that, in many cases, these ligand families expanded and diversified early in animal evolution and have been very highly conserved ever since suggests that ligand diversity is a critical feature that helps to allow this modest number of signaling pathways to exhibit the enormous range of outputs that is observed in vivo.

Paralogous ligands can have largely overlapping functions, wholly independent functions, or a complex combination of the two (Ewen-Campen *et al*. 2017). For example, in the *Drosophila* midgut, the JAK/STAT ligand *upd1* plays a critical function in intestinal stem cells (ISCs), where it promotes proliferation under basal conditions; neither *upd2* nor *upd3* is required for this function (Osman *et al*. 2012). However, when the midgut epithelium is damaged, *upd2* and *upd3* are upregulated in enterocytes and function in an additive manner to promote damage-responsive ISC proliferation (Osman *et al*. 2012). Importantly, while *upd2* and *upd3* function together in the context of midgut regeneration, in other contexts the 3 *upd* ligands have wholly separate functions, such as the role that *upd2* plays in the fat body to regulate systemic metabolism (Rajan and Perrimon 2012).

In order to tease apart the function of a pair of related genes, it is often necessary to understand their expression and coexpression patterns in space and time. The split-Gal4 system is particularly well suited to identify those cells in which 2 genes are coexpressed (Luan *et al*. 2006, 2020). In this system, a transcriptional activator domain (either VP16 or p65) and a DNA-binding domain (from Gal4), each separately fused to a leucine zipper, are separately expressed under the control of 2 different regulatory sequences. In those cells that express both components, and only in those cells, the leucine zippers bind to another to constitute a functional transcription factor that activates Upstream Activated Sequence (UAS)-based transgenes (Luan *et al*. 2006, 2020). Thus, the split-Gal4 system can be used to identify in vivo those cells that coexpress 2 genes of interest.

The split-Gal4 system has predominantly been used to generate lines driven by minimal enhancer fragments, in order to specifically label very small numbers of neurons in the brain (Tirian and Dickson 2017). However, recent studies have shown that the split-Gal4 components can also be inserted directly into a coding sequence of a gene of interest, either downstream of a T2A sequence and/or encoded in a “trojan exon,” which allows for split-Gal4 lines that recapitulate the entire expression pattern of a gene-of-interest (Diao *et al*. 2015; Ewen-Campen *et al*. 2020, 2023; Chen *et al*. 2023; Diao *et al*. 2024).

Previously, we generated a collection of knock-in split-Gal4 lines targeting the 7 Wnt ligands and demonstrated how these lines can be used to identify cells and tissues, which coexpress certain pairs of Wnt agonists in vivo (Ewen-Campen *et al*. 2020). Here, we present an expanded collection of knock-in split-Gal4 lines targeting the ligands from additional highly conserved signaling pathways in *Drosophila*: Notch, Hedgehog, FGF, EGF, TGF*β,* JAK/STAT, JNK, and PVR. These lines can be used to identify tissues that coexpress pairs of ligands-of-interest and more generally may be useful to researchers wishing to genetically manipulate specific cell populations that can be intersectionality-labeled using these reagents. Furthermore, these reagents can be used to identify potential crosstalk between different pathways by identifying cells that coexpress ligands for more than one pathway.

## Materials and methods

### Experimental animals

*Drosophila melanogaster* stocks were maintained and crossed on standard laboratory cornmeal food, with experiments performed at 25°C. We used FlyBase to find information on all genes in this study (Öztürk-Çolak *et al.* 2024). “Split-Gal4 tester lines” and UAS:2xEGFP lines were obtained from the Bloomington Drosophila Stock Center: *tub:Gal4DBD; UAS:2xEGFP* (BL60298) and *tub:VP16[AD]*, *UAS:2xEGFP* (BL60295), and UAS-2xEGFP, *w; Sp/CyO; UAS-2XEGFP* (BL60293), *w; UAS-2XEGFP; Dr/TM3,Sb* (BL60292); and *w; UAS-Stinger; MKRS/TM6b* (BL90920).

### Cloning of knock-in constructs

Coding sequences for p65-Zip^+^ and Zip^−^-Gal4DBD sequences were originally amplified from pBPp65ADZpUw (Addgene 26234; RRID:Addgene_26234) and pBPZpGAL4DBDUw (Addgene 26233; RRID:Addgene_26233), respectively, both of which were gifts of Gerald Rubin. T2A-p65-Zip^+^ and T2A-Zip^−^-Gal4DBD knock-in constructs were created using 2 previously described methods: for *upd1-3*, *Pvf1*, *Pvf3*, and all *TGFβ* ligands except *gbb*, donor constructs were generated using 1,000 bp homology arms, amplified via PCR, following the method described in the study by Ewen-Campen *et al*. (2020), with an sgRNA targeting a coding exon coinjected. For all other ligand targets, donor constructs were generated using the more efficient “drop-in” method as described in the study by Ewen-Campen *et al*. (2023) with the pUC57_Kan_gw_OK2 plasmid backbone from Kanca *et al*. (2019, 2022). Constructs in the pUC57_Kan_gw_OK2 backbone self-linearize in vivo using a synthetic sgRNA that targets either side of the donor and also encode the gene-specific sgRNA on the same plasmid.

### Transgenic *Drosophila* lines

The lines generated in this study are described in Table 1. Knock-in constructs were injected into newly laid eggs expressing germline-restricted Cas9 (*nos::Cas9*). For target genes located on the X, 2nd, or 4th chromosome, we used attP2-*nos::Cas9* (on the 3rd chromosome) as the injection stock, and for any target genes located on the 3rd chromosome, we used attP40-*nos::Cas9* as the injection stock. Injected animals were crossed to an appropriate balancer stock: X chromosome: *FM7*; 2nd chromosome: *CyO*; 3rd chromosome: *TM6b,Tb*; and 4th chromosome: *P{w[+mC] = ActGFP}unc-13[GJ]* (BL9549), and offsprings were screened for 3xP3-RFP expression. Approximately 4 independent transgenic lines were established per target gene, which were then screened for consistent expression. Each line was validated for correct insertion using either PCR genotyping, with a forward primer located 5′ to the left homology arm and a reverse primer located in the T2A sequence of the knock-in or with GFP expression in a well-characterized domain or both. All lines in this paper have been submitted to the Bloomington Drosophila Stock Center, with Research Resource Identifiers (RRIDs) given in Table 2.

**Table 1.** Lines generated in this study.

Signaling pathway	Line	p65	Gal4DBD	5′ flanking sequence
JAK-STAT	*unpaired 1 (upd1)*	-	●	CCCAGTTGCCCCACCTCGCC
	*unpaired 2 (upd2)*	●	-	AGTGGGAGAACCCGTGCGGA
	*unpaired 3 (upd3)*	●	●	TGCACCAGCAGGCGACCTTG
PVR	*PDGF- and VEGF-related factor 1 (Pvf1)*	●	●	TCCGCCAGGCGGACGATCCC
	*PDGF- and VEGF-related factor 2 (Pvf2)*	●	-	TCGCTACGTTCTCCCCCCAA
	*PDGF- and VEGF-related factor 3 (Pvf3)*	●	●	CTGAGCCTCGAACCACCAGA
TGF*β*	*decapentaplegic (dpp)*	Lethal	Lethal	-
	*glass bottom boat (gbb)*	-	●	AAACACCTCGGAGGCCGTTG
	*screw (scw)*	●	●	GTGCCACCACATATGTGACC
	*dawdle (daw)*	●	●	TAGCCCTGGTGTTGCTGGTG
	*myoglianin (myo)*	●	-	ACCGCGCTATTAGGGTTGCA
	*maverick (mav)*	●	●	ACGTGCATCACCGTTCAAAC
	*Activin-β (Actβ)*	●	●	GAAGCCAAGCCAGCAGTAGG
EGFR	*spitz (spi)*	●	●	CGTCGAGGCCTGCTCCAGCC
	*gurken (grk)*	-	●	AGATTGTTGTTCGAGCCGAA
	*Keren (Krn)*	-	●	CTGTTCCTCGCGTGCCATCG
	*vein (vn)*	●	●	GAGCAGTGAAAGCTCCGCCG
	*argos (aos)*	-	●	GCTGACCGCCGCTGCCGTTG
FGF	*pyramus (pyr)*	●	●	CGGCAAAGTGACCGCCGAGA
	*thisbe (ths)*	●	●	CAAGCGCAAGGGCCGGCGAA
	*branchless (bnl)*	●	-	GCGAAGAAACCTGCGCTTAG
NOTCH	*Delta (Dl)*	●	●	GGTAGCGGGCAGCGGAGCGG
	*Serrate (Ser)*	●	●	AAATACCAACAGCCATCTAC
HH	*Hedgehog (hh)*	●	●	ACCTCTGGAGGGTGTGATCC
TNF-*α*	*eiger (egr)*	●	●	GCAGACAACGCGTGTATCGC

●, line generated; -, line not generated; PDGF, platelet-derived growth factor; VEGF, vascular endothelial growth factor.

**Table 2.** Resource identifiers for lines generated in this study.

Line	RRID
aos-Gal4DBD	RRID:BDSC_605184
bnl-p65	RRID:BDSC_605185
Dl-Gal4DBD	RRID:BDSC_605202
Dl-p65	RRID:BDSC_605201
egr-Gal4DBD	RRID:BDSC_605187
egr-p65	RRID:BDSC_605186
gbb-Gal4DBD	RRID:BDSC_605188
hh-Gal4DBD	RRID:BDSC_605190
hh-p65	RRID:BDSC_605189
Pvf2-p65	RRID:BDSC_605191
pyr-Gal4DBD	RRID:BDSC_605200
pyr-p65	RRID:BDSC_605199
Ser-Gal4DBD	RRID:BDSC_605193
Ser-p65	RRID:BDSC_605192
spi-Gal4DBD	RRID:BDSC_605195
spi-p65	RRID:BDSC_605194
ths-Gal4DBD	RRID:BDSC_605197
ths-p65	RRID:BDSC_605196
vn-Gal4DBD	RRID:BDSC_605198
vn-p65	RRID:BDSC_605688
Remaining lines	In progress

### Antibody staining

Tissues were dissected in PBS, fixed for ∼30 min in 4% paraformaldehyde in PBS, permeabilized in PBS containing 0.3% Triton-X, and stained using standard protocols. Tissues were stained for GFP using Alexa488-coupled anti-GFP (Invitrogen A21311, used at 1:400) and DAPI to visualized nuclei. For larval body wall muscle, a muscle relaxant buffer was used during dissection (5 mM EGTA and 5 mM MgCl_2_ in PBS) prior to fixation. Larval muscle was visualized using Alexa555-coupled phalloidin. Sagittal sections of whole adult flies were performed and visualized as described in the study by Ewen-Campen *et al*. (2023). Confocal imaging was performed on a Zeiss Axio Observer Z1 with a LSM980 Scan Head, part of the Microscopy Resources on the North Quad (MicRoN) core at Harvard Medical School.

No statistical analyses were performed in this study.

## Results and discussion

### A resource of split-Gal4 knock-in lines targeting genes encoding conserved signaling ligands

**Fig. 1.**
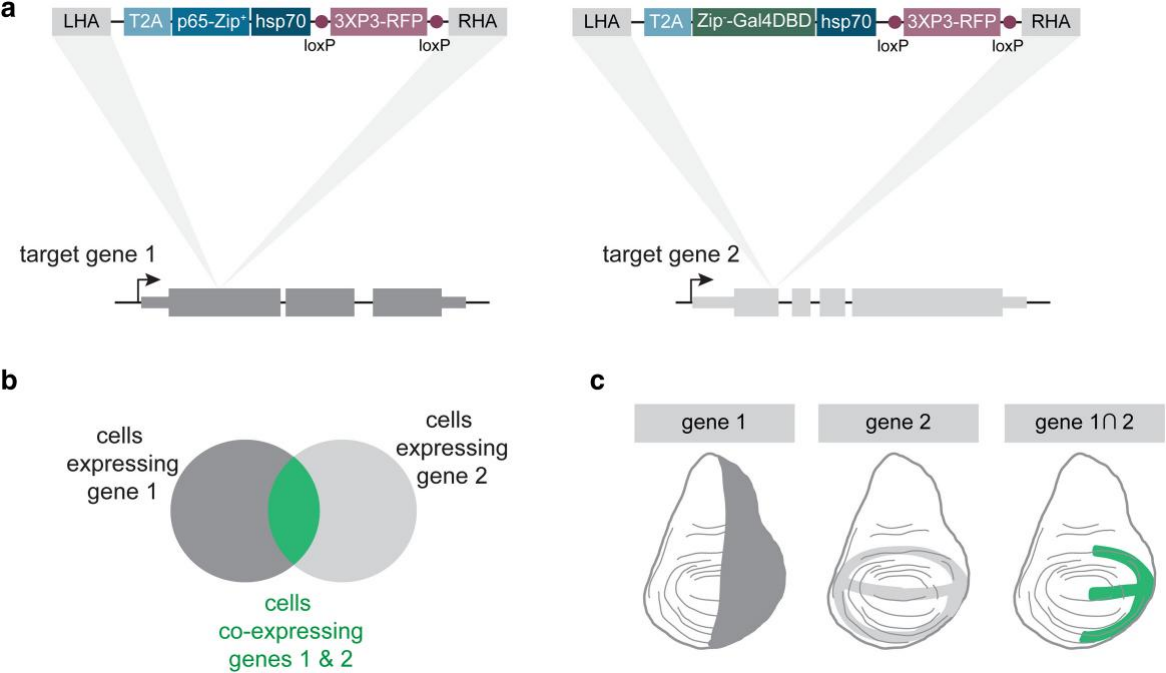
Strategy for creating split-Gal4 knock-in lines for conserved signaling ligands. a) Knock-in cassettes containing T2A-p65 or T2A-Gal4DBD were inserted using CRISPR-Cas homology-directed repair into a coding exon of each signaling ligand, so that the expression domain of each split-Gal4 line captures the native expression pattern of the target gene. b) Schematic of the split-Gal4 system. Only in those cells that coexpress both target genes are the 2 split-Gal4 components reconstituted via leucine zipper binding into a functional transcription factor. c) Cartoon example of 2 hypothetical split-Gal4 lines in a developing wing disc, demonstrating how the expression of a UAS-based transgene is limited to only those cells that coexpress both gene 1 and gene 2. Dorsal is up, anterior is left. LHA, left homology arm; RHA, right homology arm; 3xP3-RFP, fluorescent marker for transgenesis.

We created CRISPR knock-in donor plasmids to insert either T2A-p65 or T2A-Gal4DBD into each of the ligands shown in Table 1. We designed these donor constructs to be inserted in a coding exon (Fig. 1a), targeting shared exons for any target gene with multiple isoforms. These inserts target coding exons and therefore are presumed to produce loss-of-function alleles of the target gene. These knock-in constructs also contain a 3xP3-RFP fluorescent marker used to recover transformants, and this marker is flanked by loxP sites so that it can be removed in the future by crossing to a line containing a source of *Cre*. When a p65 knock-in line is crossed to a Gal4DBD knock-in line, those cells that coexpress both ligands can be visualized using a UAS-based reporter, and this population of cells can be genetically manipulated (Fig. 1b and c).

For each target gene, we first confirmed that the insertion segregates on the expected chromosome and then validated that the insertion was correct using PCR-based genotyping and sequencing, recapitulation of a known *UAS:GFP* expression pattern, or both.

We successfully recovered 39 transgenic lines: both p65 and Gal4DBD for 15 genes (totaling 30 lines) and either p65 or Gal4DBD knock-ins for 9 additional genes (Table 1). We were unable to recover any knock-ins for *dpp*, despite multiple attempts at the extreme 3′ end of the coding sequence, which is consistent with the fact that this gene is a dominant lethal (Wharton *et al*. 1993; Table 1). Altogether, this collection should allow for analysis of nearly all pairwise combinations of ligands within a signaling family and many combinations across pathways.

To determine whether our knock-in lines successfully recapitulate the expression pattern of the genes into which they are inserted and whether the T2A-p65 and T2A-Gal4DBD lines mirror one another for a given target, we examined their expression patterns in L3 larval wing discs. We crossed each of our knock-in lines to a reciprocal “tester” line, containing either *tub:VP16-AD* or *tub:Gal4DBD* and a *UAS:2xEGFP* reporter. We focused on those genes for which we obtained both p65 and Gal4DBD lines and those genes predicted to be expressed in a specific pattern in wing discs.

**Fig. 2.**
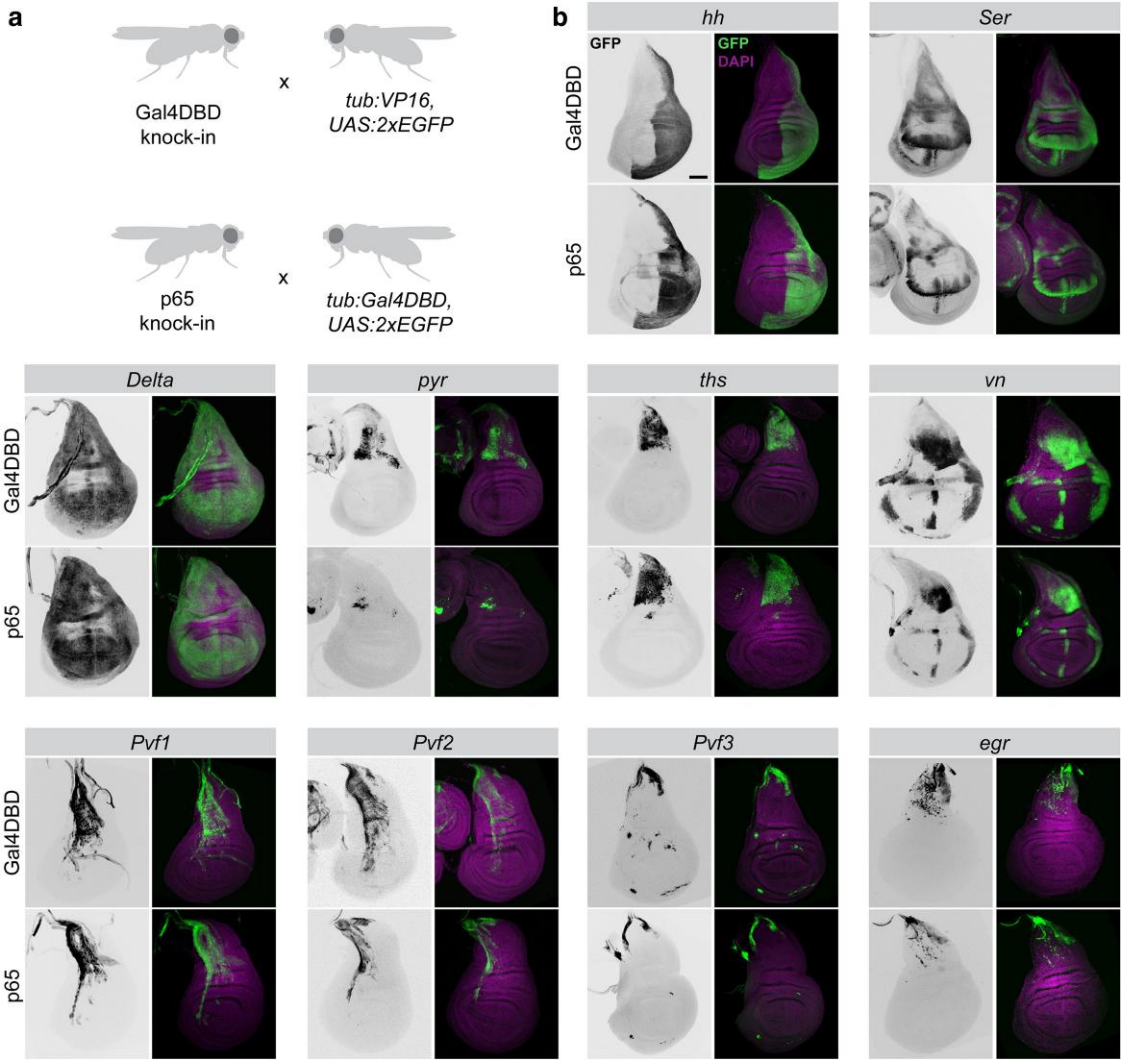
Expression patterns for a selection of split-Gal4 lines in the L3 wing disc. a) The full expression pattern of each knock-in line was determined by crossing to a ubiquitously expressed split-Gal4 “tester line”: *tub:VP16*, *UAS:2xEGFP* for Gal4DBD knock-ins and *tub:Gal4DBD*, *UAS:2xEGFP* for p65 knock-ins. b) For each target gene, transgenic lines expressing Gal4DBD (top row) or p65 (bottom row) are shown. Dorsal is up, anterior is left.

The split-Gal4 lines generated in this study successfully capture the endogenous expression pattern of their target genes, and we generally observed that p65 lines and Gal4DBD for a given target gene were essentially congruent (Fig. 2). We found that both p65 and Gal4DBD lines targeting *hh* drive expression in the posterior compartment of the wing disc (Lee *et al*. 1992; Tabata *et al*. 1992; Fig. 2) and that lines targeting the Notch ligands *Delta* and *Ser* recapitulate well-characterized expression patterns (Doherty *et al*. 1996; Yan *et al*. 2004). Lines targeting the FGF ligands *pyr* and *ths* are both expressed in the epithelial cells of the notum, overlaying the adult muscle precursors, as previously reported (Everetts *et al*. 2021; Patel *et al*. 2022; Fig. 2). *vn*-*p65/Gal4DBD* lines mirror endogenous patterns of *vn* expression (Simcox *et al*. 1996).

The expression patterns of other ligands are not well characterized in the wing disc, but several lines of evidence suggest that the expression patterns we observe mirror endogenous gene expression patterns. For example, to our knowledge the expression patterns of *Pvf1*, *Pvf2*, and *Pvf3* have not been characterized in the wing disc, but we observed that *Pvf1* and *Pvf2* expressions were restricted to the apical structures of the disc, as described for *PVF1* protein (Rosin *et al*. 2004), and enriched primarily in the notum, and that *Pvf3* expression was primarily found at the dorsal edge of the notum and the stalk, in a location where the *PVR* receptor is known to function in dorsal closure (Ishimaru *et al*. 2004). These expression patterns were also consistent with in silico predictions from single-cell RNAseq (scRNAseq) studies of the wing disc (Deng *et al*. 2019; Worley *et al*. 2022). Relatedly, the JNK ligand *egr* is also enriched near the stalk and notum, where the JNK target gene *puc* is endogenously expressed (Agnes *et al*. 1999) and where in silico predictions indicate *egr* is enriched (Deng *et al*. 2019).

While the expression patterns between p65 and Gal4DBD lines were largely similar, we observed that in some cases, the expression of our Gal4DBD lines was notably stronger than the p65 lines targeting the same ligands (Fig. 2, see e.g. *Ser*, *pyr*, and *vn*). We noted that these differences could be caused by differential strengths of the 2 “tester” lines we used; specifically, the *tub:Gal4DBD, UAS:2xEGFP* tester line may drive weaker expression than the *tub:VP16-AD, UAS:2xEGFP* line.

**Fig. 3.**
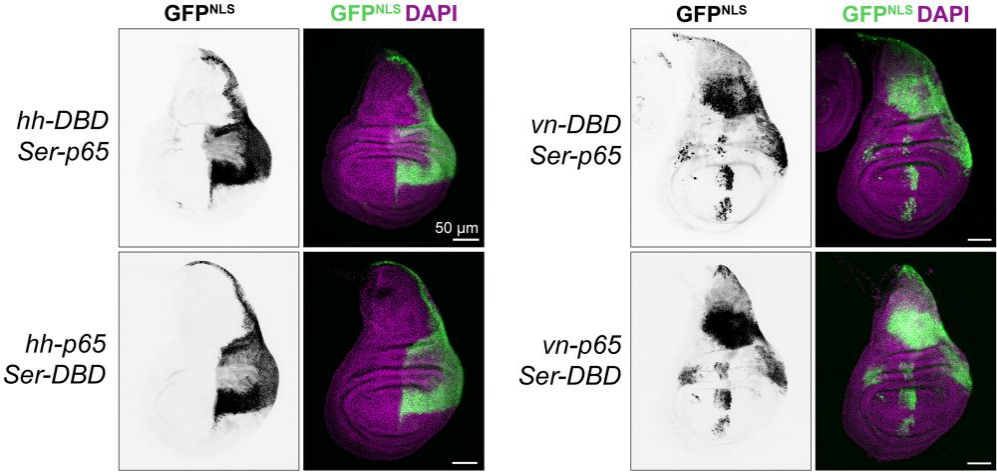
Intersectional labeling by split-Gal4 lines. Expression patterns in the L3 wing disc of *hh ∩ Ser* and *vn ∩ Ser*, in each possible p65/Gal4DBD configuration. Nuclear-localized UAS:GFP^Stinger^ was used in these experiments. Dorsal is up, anterior is left.

To test whether this was the case, we crossed a variety of our knock-in split-Gal4 lines to one another, in both directions. We examined expression of *hh-p65 ∩ Ser-Gal4DBD* and *hh-Gal4DBD ∩ Ser-p65* and performed similar bidirectional crosses between *vn ∩ Ser*. In each case, we observed essentially indistinguishable expression patterns regardless of which gene was driving p65 vs Gal4DBD, indicating that these lines drive very similar expression levels to one another (Fig. 3) and indicating that the *tub:VP16-AD*, *2xEGFP* tester line likely drives lower expression than the *tub:Gal4DBD*, *UAS:2xEGFP* line. Altogether, these results demonstrate that these knock-in split-Gal4 lines successfully recapitulate the expression patterns of their target genes and that the expression levels of p65 and Gal4DBD knock-in lines are very similar to one another.

### TGF*β* ligands *maverick* and *myoglianin* are coexpressed broadly in larval and adult muscles

To demonstrate possible applications for this collection of split-Gal4 lines, we first turned to the 7 TGF*β* family ligands. Using the bioinformatic tool Paralog Explorer (https://www.flyrnai.org/tools/paralogs/web/) (Hu *et al*. 2022), we queried the coexpression correlation of each of the 7 TGF*β* ligands against the others across 4 high-throughput RNAseq datasets from the modENCODE project: across tissues, across cell lines, across developmental datasets, and across experimental treatments. This analysis suggested that *maverick (mav)* and *myoglianin (myo)* are consistently among the TGF*β* ligands with the most highly correlated coexpression across all 4 categories of comparison.

*myo* and *mav* are both located on the 4th chromosome. Along with *Actβ* and *daw*, *myo* is a member of the activin family of ligands (Lo and Frasch 1999; Upadhyay *et al*. 2017) and is orthologous to mammalian GDF11 and myostatin (Demontis *et al*. 2014). In contrast, *mav* does not fall neatly into either the activin or Bone Morphogenic Protein families (Nguyen *et al*. 2000; Upadhyay *et al*. 2017), and phylogenetic analysis of TGF*β* ligands has not definitively identified which other TGF*β* ligand is most closely related to *mav* (Zee *et al*. 2008). Loss-of-function *myo* mutants are pupal lethal, and this ligand has been shown to be expressed in both glia and in muscles where it plays a number of critical signaling roles (Awasaki *et al*. 2011; Demontis *et al*. 2014; Augustin *et al*. 2017; Upadhyay *et al*. 2020)*. mav* loss-of-function mutants are viable but display neurological defects (Myers *et al*. 2018; Hoyer *et al*. 2018).

**Fig. 4.**
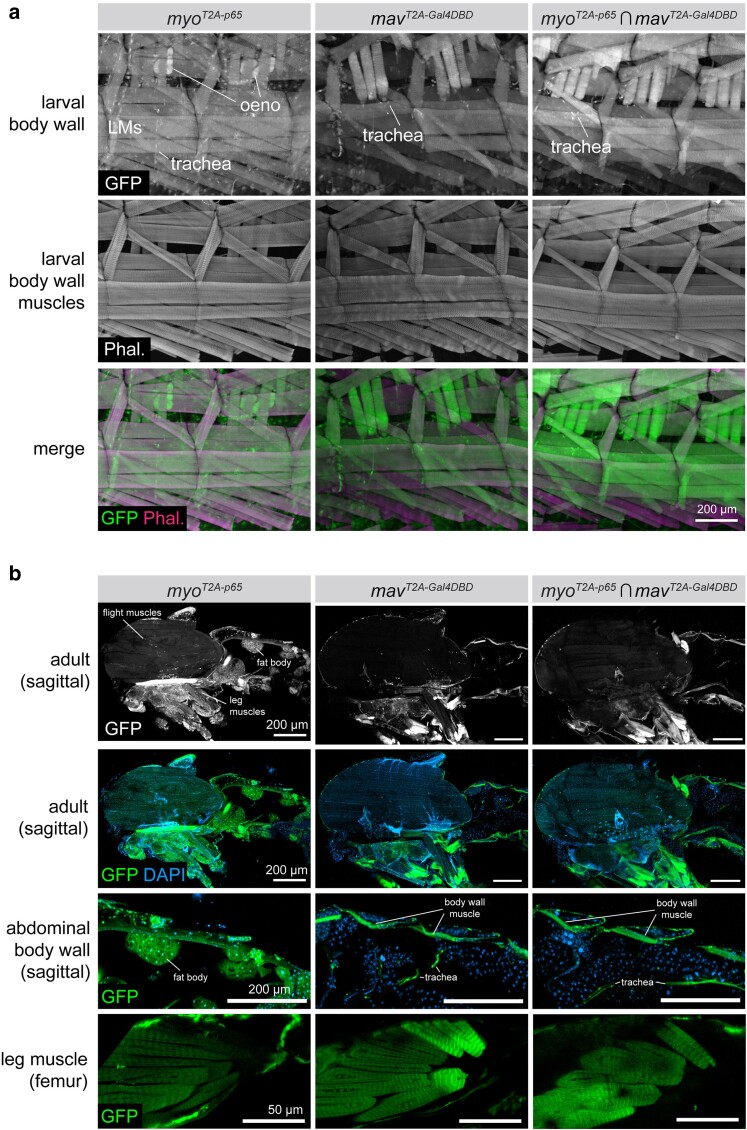
The TGF*β* ligands *maverick* and *myoglianin* are coexpressed in muscles of the larva and adult. a) Expression of *myo-p65* and *mav-Gal4DBD* and coexpression of *myo-p65 ∩ mav-Gal4DBD* in L3 larval body wall tissues, including muscles, trachea, and oenocytes. Both ligands are expressed broadly in nearly all larval body wall muscles. b) Expression of *myo-p65*, *mav-Gal4DBD*, and *myo-p65 ∩ mav-Gal4DBD* in sagitally sectioned adult carcass (top 3 rows) and in adult leg muscles (bottom row). LM, larval muscles; Phal, phalloidin; oeno, oenocytes.

Given the high degree of *myo* and *mav* coexpressions predicted in silico, we examined their coexpressions in vivo. In L3 larva, we found that *myo-p65* was expressed broadly in all muscles of the body wall, as well as detectable in oenocytes and trachea (Fig. 4a). *mav-Gal4DBD* was detected in larval muscles and in trachea (Fig. 4a), and intersectional labeling of *myo ∩ mav* was detected in all larval muscles and in trachea (Fig. 4a). In adults, *myo ∩ mav* coexpression was again detected in all muscles of the body wall and the legs but largely excluded from the flight muscles of the thorax (Fig. 4b). As in larva, *myo-p65* expression was broader than *mav-Gal4DBD* and could be detected in fat body, salivary gland, and other tissues, whereas *mav-Gal4DBD* appeared largely restricted to the muscles and some trachea (Fig. 4b.) Taken together, these results suggest that *myo* and *mav* are broadly coexpressed in both muscles and trachea throughout larval and adult life and that future studies may benefit from double knock-out experiments to examine the possibility of overlapping function.

As a second example of how this collection of split-Gal4 lines can be used, we examined the coexpression of JAK/STAT ligands in the adult gut, where all 3 *upd* ligands play important functions (Jiang *et al*. 2009; Osman *et al*. 2012). All 3 ligands are upregulated in the midgut in response to tissue damage, with *upd3* showing the highest levels of damage-responsive upregulation (Jiang *et al*. 2009; Osman *et al*. 2012), and it has been shown previously that *upd2* and *upd3* have an additive effect on gut response to damage, with *upd2*, *upd3* double mutants showing stronger effects than either of the single mutants (Osman *et al*. 2012). However, it is not known whether the same cells in the gut upregulate *upd2* and *upd3* or whether they are upregulated in distinct gut cell populations.

**Fig. 5.**
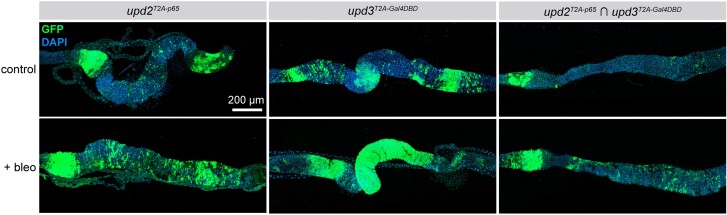
The JAK/STAT ligands *upd2* and *upd3* are coexpressed in cells of the posterior midgut in homeostasis and in response to cellular damage. Expression of *upd2-p65* ∩ *tub:Gal4DDBD* and *upd3-Gal4DBD* ∩ *tub:VP16* and coexpression of *upd2-p65 ∩ upd3-Gal4DBD* in the adult posterior midgut in control guts (top row) and guts damaged by bleomycin treatment (bottom row). Anterior is to the left.

We first examined the expression of *upd2-p65* and *upd3-Gal4DBD* in control midguts and in midguts of flies which had been damaged by bleomycin feeding, by crossing these lines to the appropriate *tub-*driven split-Gal4 tester line. As expected, both *upd2* and *upd3* were strongly upregulated by bleomycin-induced damage (Fig. 5), with *upd3* showing dramatically stronger upregulation (Fig. 5). When we examined the intersectional staining of *upd2 ∩ upd3*, we observed a notable increase in the population of GFP+ cells in the midgut, indicating that many individual cells in the midgut simultaneously upregulate the expression of *upd2* and *upd3* in response to damage, whereas many other cells must upregulate *upd3* alone given the differential expression pattern of *upd3-Gal4DBD* vs *upd2 ∩ upd3.* Altogether, these experiments demonstrate that our collection of split-Gal4 lines faithfully recapitulates dynamic expression changes in response to tissue damage and can additionally be used to identify those cells that specifically upregulate pairs of genes simultaneously.

Previously, identifying the cells or tissues that coexpress a pair of genes-of-interest required such techniques as double antibody staining or double in situ hybridization. The creation of intersectional genetic labeling systems such as split-Gal4 system provided a genetic means to specifically label such cells and has the additional benefit that these cells can be genetically manipulated in addition to simply being labeled. Here, we describe a collection of split-Gal4 lines targeting a collection of highly conserved signaling ligands, many of which belong to closely related paralogs. We demonstrate that these lines recapitulate the endogenous expression patterns and that they can be used to identify cells or tissues where genes-of-interest are coexpressed.

The resource described here, together with our previous collection of knock-in split-Gal4 lines targeting the Wnt family of ligands (Ewen-Campen 2020), will allow detailed analysis of most major signaling pathway ligand expression and coexpression in *Drosophila*. We believe these reagents will be useful to others in the field and have made them available at the Bloomington Stock Center.

We also note that these reagents may be useful for the annotation of scRNAseq datasets. As we and others have previously shown, split-Gal4 can be a powerful technique to map in silico cluster predictions to in vivo anatomy because most clusters cannot be uniquely identified using a single marker gene (Chen *et al*. 2023; Ewen-Campen *et al*. 2023). Given the widespread expression of many signaling ligands in multiple tissues throughout development and in the adult, the lines described here may prove useful tools to locate and study cell types first identified via scRNAseq.

## Data Availability

All data necessary for confirming the conclusions of this article are present within the article, figures, and tables, and all transgenic lines are available or will soon be available from the Bloomington Drosophila Stock Center.
